# The time course of visuo-spatial working memory updating revealed by a retro-cuing paradigm

**DOI:** 10.1038/srep21442

**Published:** 2016-02-12

**Authors:** Daniel Schneider, Christine Mertes, Edmund Wascher

**Affiliations:** 1 Leibniz Research Centre of Working Environment and Human Factors, TU Dortmund, Germany

## Abstract

We investigated the influence of non-cued working memory representations on further information processing. A retro-cue indicated if items on the left or right side of a previous memory array remained relevant. Subsequently, a central probe item was presented with a varying stimulus onset asynchrony (SOA: 300, 400, 600, 1000, 1800 ms). Participants had to state whether this stimulus was shown on the cued side of the memory array. The probe was either a cued, non-cued or new item. Non-cued probes were associated with delayed response times and an increased frontal negativity from 400–600 ms indicating a higher processing conflict compared to new probes. These effects were strongest for the 300 and 400 ms SOAs and decreased in longer SOA conditions, pointing toward a benefit when there was sufficient time for working memory updating. Furthermore, contralateral negativities at posterior (PCN) and anterior sites (ADAN) reflected the attentional orienting toward cued information while selective retention was associated with a sustained suppression of posterior induced alpha power contralateral to retro-cue direction. Results suggest that retro-cue induced updating of visuo-spatial working memory requires about 500 ms to transfer the non-cued contents into a passive and fragile short-term memory state with less impact on ongoing information processing.

Working memory (WM) can be defined as a set of functions that enable the temporary maintenance of information and the access to this information in order to perform higher-level cognitive operations^1^,^2^. To support goal-directed behavior, the WM resources available must be provided for relevant information while the passage of irrelevant inputs into WM has to be inhibited. In a first place, selective attention serves as a filter for the information represented in WM. Furthermore WM has to be updated to provide resources for new and potentially relevant information. The present study focuses on the fate of representations in WM that are no longer required following WM updating. This is investigated by means of a so-called retroactive cuing (retro-cuing) paradigm and event-related parameters of the electroencephalogram (EEG).

Incoming visual information is initially transferred into a sensory memory store referred to as iconic memory^3^. Iconic memory only lasts about 500 ms and is subject to interference by newly incoming visual signals. Selective attention can guide these fragile iconic representations into a longer lasting and more stable store that can be referred to as WM. The active maintenance of information in WM is thus related to the focusing of attention on a subset of initially available mental representations^4^,^5^. In line with this account, current studies have revealed that selective attentional mechanisms are also involved in the updating of visuo-spatial WM. Change detection or recognition tasks based on WM were combined with spatial cues that were presented after an initial memory array. Comparable to spatial cues presented prior to memory encoding, these retroactive cues (retro-cues) led to a shift of attention toward the cued items in WM. This shift of attention was associated with an increase in task performance that was not found when spatially neutral retro-cues were presented^6^,^7^,^8^,^9^,^10^.

Retroactive focusing of attention on mental representations was therefore related to protection from decay over time and from interference with newly incoming information^9^,^11^,^12^,^13^. However, the fate of non-cued WM representations following retro-cue presentation is not yet resolved. A spatial retro-cue might cause the inhibition or passive decay of non-cued WM representations leading to a drop of information from memory. Alternatively, recent studies suggest that WM updating can be seen as a more flexible mechanism. Following a retro-cue, non-attended or non-cued information is transferred to a more passive and fragile visual short-term memory (vSTM) state^14^,^15^,^16^. New visual inputs presented at the same location and consisting of comparable objects can interfere with this representation. Thus, fragile vSTM contains both object-specific and location-specific information^14^. Recent research has shown that representations in fragile vSTM can still conflict with processing of attended mental representations^17^. Correspondingly, a study by van Moorselaar *et al*.^15^ revealed that non-attended vSTM representations could again be attended or re-activated by means of a second retro-cue. Thus, WM representations declared as irrelevant are not lost completely but transferred to a different memory state.

Event related potentials (ERPs) of the EEG can be used to map the neural mechanisms that are involved in these retro-cue induced updating processes. Contralateral delay activity (CDA^18^) is characterized as an increase in activation at posterior electrodes contralateral compared to ipsilateral to selectively memorized information of a bilateral stimulus array. As CDA was shown to be associated with the amount of information activated in visuo-spatial WM, it can also be used to study the modulation of WM contents when retro-cues led to a change of relevance of the maintained information^7^,^19^. However, Schneider *et al*.^17^ revealed that ERP asymmetries referred to lateralized retro-cues were not sustained throughout the retention interval when the target information was presented non-lateralized. Rather, a posterior contralateral negativity (PCN) appeared 200–400 ms after retro-cue onset and was followed by a reversed asymmetry indicating the re-allocation of attention to the to-be-probed location (i.e. distractor positivity or Pd). While PCN was associated with the selection of cued mental representations, a later contralateral suppression in induced alpha power indicated that the cued information was retained in WM^17^.

Although these electrophysiological correlates depict the time course of the updating of visuo-spatial WM, they give no direct information about the fate of the non-cued WM contents^20^. The present study approaches this issue by varying the stimulus onset asynchrony (SOA) between retro-cue and memory probe onset. The task included a four-item memory array with differently colored circles on the left and right side of fixation. Subsequently, a spatial retro-cue indicated if left or right items remained relevant for a later recognition task. The following retention interval could vary between five different time intervals (i.e. 300, 400, 600, 1000 and 1800 ms). It was recently shown that the handling of non-cued mental representations depends on retro-cue validity^21^. Therefore, retro-cues in the current task were always reliable in indicating the to be probed memorized information (see Fig. 1). Following the retro-cue, a display with a non-lateralized probe stimulus contained either a stimulus equal to a memory item on the cued side (cued probe condition) or non-cued side of the memory array (non-cued probe condition) or an item that was new with respect to the initial memory array (new probe condition). Participants were instructed to state whether the probe item was previously part of the cued memory array items (YES response for cued probe condition; NO response for non-cued and new probe conditions). Schneider *et al*.^17^ revealed that this procedure ensured the focusing on the cued mental representations to maximize performance. All probe conditions were solved by comparing the probe with the cued mental representations, while residual representations of non-cued information interfered with this comparison process in the non-cued probe condition.

The experimental design allows the investigation of two important research questions. Firstly, changing the length of the retention interval prior to probe presentation should give new insights about the neural mechanisms underlying retro-cue induced WM updating. A recent study by Myers *et al*.^22^ suggested that a posterior contralateral alpha suppression following a retro-cue reflects a transient re-orienting to the cued information. However, the authors did not vary the length of the retention interval following the retro-cue. We hypothesize that the contralateral decrease in alpha power observed in prior retro-cue studies rather reflects the selective maintenance of cued mental representations in WM^17^,^23^. If this is the case, respective posterior asymmetries in alpha power should persist in the varying retention intervals (see five SOA conditions). The duration of the PCN effect should not vary as a function of the retention interval because PCN was associated with attentional mechanisms preceding the selective retention of cued mental representations. Secondly, the different SOA conditions enable us to investigate how non-cued mental representations change over time. Compared to prior studies on the fate of non-cued mental representations in WM (e.g.^13^,^21^), the current paradigm enables us to draw a time course of visuo-spatial WM updating based on retro-cues that are always reliable in indicating the to-be-probed information. For this purpose, we compared behavioral performance and electrophysiological data between the non-cued and new probe conditions. While the same behavioral response is required (NO response), only non-cued probes but not new probes are initially encoded in WM. Depending on their representational strength, non-cued probes should be associated with a stronger familiarity signal than new probes. This led to a conflict with the negative decision (i.e. NO response) during probe processing that was associated with higher response times (RTs) and an increased frontal negativity starting at about 400 ms following memory probe onset (i.e. N450^17^,^24^). However, the SOA variation should critically modulate the strength of the non-cued mental representations, because the ability to update WM should depend on the time available after retro-cue and before probe onset. We therefore hypothesize that the RT difference between the non-cued and new probe conditions decreases the longer the retention interval following the retro-cue. This should in the same way be reflected by an SOA-dependent decrease of the N450 effect. Studying the SOA effect on these behavioral and electrophysiological parameters will allow for drawing conclusions both about the time required to drop non-cued information from WM and about the time these more passive representations of non-cued information still affect ongoing information processing.

## Results

### Behavioral results

RTs and response accuracy were analyzed by means of 3 × 5 analyses of variance (ANOVAs) including the within-subject factors “probe condition” (cued, non-cued, new) and “SOA” (300, 400, 600, 1000, 1800 ms). Accuracy varied with probe condition, F(2,36) = 14.77, ε = 0.58, p < 0.001, 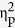 = 0.45. Percent correct responses were lowest in the cued probe condition compared to the non-cued condition (p < 0.001) and new condition (p < 0.001). Additionally, accuracy was higher in the new probe condition compared to the non-cued probe condition (p < 0.05). Also the factor SOA had an effect on task accuracy with lowest performance in the 300 ms and 400 ms conditions, F(4,72) = 8.67, p < 0.001, 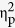 = 0.33 (see Fig. 2A). However, SOA did not further modulate the effect of probe condition on accuracy, F(8.144) = 1.24, ε = 0.55, p = 0.3, 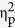 = 0.06.

RTs varied with probe condition, F(2,36) = 33.63, ε = 0.58, p < 0.001, 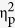 = 0.65. Slowest RTs were observed in the non-cued probe condition compared to the new probe condition (p < 0.001) and cued probe condition (p < 0.001). RTs were also modulated by SOA, F(4,72) = 64.54, ε = 0.38, p < 0.001, 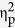 = 0.78, with slowest RTs observed for short SOAs (see Fig. 2B). Additionally, the effect of SOA on RTs differed between probe conditions, F(8,144) = 5.25, p < 0.001, 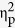 = 0.23. To test if there was a decline in RT difference between the non-cued and new probe condition with increasing SOA, an ANOVA with RT difference as dependent variable and SOA as continual/numeric factor was conducted. As shown in Fig. 2C, the RT difference between the non-cued and new probe conditions (NO response correct in both conditions) revealed an overall decline with increasing SOA, F(1,18) = 29.55, p < 0.001, 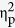 = 0.62. Post-hoc analyses showed that while the RT difference did not change between the 300 ms and 400 ms SOAs (p = 0.32), it was smaller for the 600 ms SOA compared to the 400 ms SOA (p < 0.01). Again, while RT differences did not diverge between the 600 ms and 1000 ms SOAs (p = 0.95), a weaker effect was revealed for the 1800 ms SOA compared to these SOA conditions (p-values <0.05).

### Electrophysiological results

#### Retro-cue analyses

Figures 3 and 4 show the contralateral vs. ipsilateral ERPs referred to the retro-cue for the 600 ms, 1000 ms and 1800 ms SOA conditions. The topographic maps in the PCN time window (250–300 ms) confirmed a posterior maximum (see Fig. 3). Additionally, there was a more anterior contralateral negativity over pre-motor areas that overlapped with PCN (see Fig. 4). In line with studies on the attentional mechanisms following spatial cues, this effect was labeled anterior directing attention negativity or ADAN^25^,^26^,^27^. No sustained asymmetry was observed in the interval from retro-cue to probe presentation but subsequent to PCN/ADAN a reversed effect with increased negativity ipsilateral compared to contralateral to the cued memory items was shown. According to studies that investigated the neural mechanisms underlying the attentional orienting to irrelevant spatial cues presented prior to a target display, we labeled this component distractor positivity or Pd^28^,^29^,^30^. The Pd effect was again limited to posterior electrodes and was strongest over parieto-occipital brain regions (see Fig. 3).

ERP effects were assessed by measuring the negative or positive area under the contralateral minus ipsilateral difference wave. To confirm statistical significance of these effects, a distribution of area values that would be expected if there were only random lateralized activation in the data was calculated. This was achieved by randomly assigning the side of the retro-cue for each trial. If the measured area value concerning the contralateral minus ipsilateral difference waves was higher than 95% of the values received within the random distribution, a significant difference from chance level was assumed (for a detailed explanation of the procedure see the methods section).

Randomization tests revealed a significant negative deflection in the contralateral vs. ipsilateral difference curve at PO7/8 (PCN) and FC3/4 (ADAN) in the 200–500 ms time window. Also the positive area under the contralateral minus ipsilateral difference curve in an interval from 400–700 ms (i.e. Pd) was significant (see Figs 3 and 4).

As an indication of alpha lateralization (PO7/PO8) the contralateral and ipsilateral portions of induced power were measured in the 10–14 Hz range for the 600 ms, 1000 ms and 1800 ms SOA conditions (see Fig. 5). Only these SOA conditions were used because there was a strong overlap of retro-cue processing and probe processing in the 300 ms and 400 ms SOA conditions (for more details regarding the time-frequency analysis see the methods section). There was a significant decrease in induced posterior alpha power in all SOA conditions (see Fig. 5). For the 600 ms SOA condition, the contralateral alpha power decrease was revealed in an interval from 300 ms to 600 ms after the retro-cue, t(18) = −4.1, p < 0.001, d = −1.33. A comparable effect was also shown in the 300 ms to 1000 ms interval for the 1000 ms SOA condition, t(18) = −3.2, p < 0.01, d = −1.04, and 1800 ms SOA condition, t(18) = −4.03, p < 0.001, d = −1.31. Figure 5 shows that there was still an alpha power asymmetry in a later phase of the retention interval in the 1800 ms SOA condition (i.e. 1400–1800 ms). Yet, this contralateral decrease in alpha power failed to reach statistical significance, t(18) = −1.24, p = 0.11, d = −0.4.

#### Probe analyses

ERPs time-locked to probe display onset were analyzed by calculating the difference of the ERPs in the non-cued and new conditions. The negative area under this differences curve was measured in the time window from 400–600 ms to determine the N450 effect at electrodes FCz, FC1, Fz and F3. The analysis revealed a stronger frontal negativity for the non-cued compared to the new probe condition (i.e. the N450 effect). Figure 6 shows that the mean negative area under the non-cued minus new probe condition difference curve in the 400–600 ms interval declined with increasing SOA, F(1,18) = 5.38, p < 0.05, 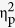 = 0.23. The topographies of the difference curves in a time window from 400–550 ms indicate that the 300 ms and 400 ms SOA conditions featured the strongest negative complex in these difference curves with maxima over midline-frontal and left-frontal areas. Comparable to the procedures run for PCN, Pd and ADAN, the N450 effect was also tested for statistical significance for each SOA condition. The resulting threshold values (95% and 90% of the random distribution) are given in Fig. 6. While for the first four SOA conditions (300, 400, 600 and 1000 ms) the N450 effect differed significantly from chance (marginally significant result for 1000 ms SOA condition), there was no longer a significant N450 difference for the 1800 ms SOA condition (see Fig. 6).

## Discussion

The present study investigated the neural mechanisms underlying retro-cue induced WM updating and the fate of non-cued representations. The retro-cues used in the current study were always reliable in indicating the relevant information for a later recognition task. Memory probes matched a color of the cued WM items (cued probe condition) or non-cued items (non-cued probe condition). Additionally, the probe could be displayed in a color that was not included in the prior memory array (new probe condition). To study the time course of WM updating and the fate of non-cued mental representations, the interval between retro-cue and probe display onset was varied in five steps (300, 400, 600, 1000 and 1800 ms SOA).

In explaining the performance differences between the cued and the non-cued/new probe conditions, it has to be noted that the current experimental design was exclusively based on retro-cue trials. Schneider *et al*.^17^ used a comparable experimental design further including spatially neutral retro-cues and revealed a retro-cue benefit (selective vs. neutral) both for accuracy and RTs. Furthermore, ERP asymmetries time-locked to the probe display indicated that all probe conditions following a selective retro-cue were solved by means of a comparison processes based on the cued information^17^. Respective ERP results for the current experiment are shown in Supplementary Figure S1. Thus, in order to respond adequately to each probe condition, attention had to be re-oriented toward the cued information. This indicates that the faster RTs for cued probes compared to non-cued and new probes were not based on a probe condition specific drawing on the memory array information (cued vs. non-cued information). Instead, the RT benefit for the cued condition should be related to a general speed advantage for YES responses (or “same” responses referred to the cued information) compared to NO responses (or “different” responses referred to the cued information) that usually appears in recognition tasks with unbiased response instructions^31^. The decreased accuracy for the cued probe condition compared to the non-cued and new probe conditions (see Fig. 2A) should be related to the inability of some participants to initially store four memory array items in WM. When the probe color matched a color that had not been encoded, participants should have been more prone to respond with “NO”. While this is an erroneous response in the cued probe condition, it is correct when probes correspond to non-cued or new items^17^. A further explanation for the lower accuracy and faster RTs in the cued probe condition might be a speed-accuracy trade-off. Participants might have responded faster at the cost of higher error rates. However, such a strategic approach was rather unlikely, because participants were not explicitly informed about the three different probe conditions, but were solely instructed to judge as fast and accurate as possible if the probe color matched one of the cued colors in the memory array.

Furthermore, the current data support the notion that the familiarity signal induced by the non-cued information conflicts with the negative decision. Longer RTs (see Fig. 2B) and lower response accuracy were revealed for the non-cued probe condition compared to the new probe condition. However, this RT effect was reduced with increasing SOA (see Fig. 2C), suggesting that the processing conflict in the non-cued probe condition became weaker as more time passed between presentation of the retro-cue and probe display. Interestingly, the strongest SOA effect on the RT difference between the non-cued and new probe condition appeared between 400 and 600 ms. While the RT difference for the 300 ms and 400 ms SOA conditions added up to 60 ms, the effect was reduced to 37 ms in the 600 ms SOA condition (see Fig. 2C). This is in line with current notions about the impact of retro-cues on the retention of visuo-spatial information in WM. Retro-cues lead to a focusing of active WM retention on the cued mental representations^8^,^32^.

The electrophysiological data were used to map the time course of these WM mechanisms. PCN was associated with the orienting of attention according to retro-cue direction. This accords with studies showing a contralateral negativity over parieto-occipital areas for the attentional orienting toward relevant perceptual information^33^,^34^,^35^,^36^,^37^,^38^. Yet, sensory asymmetries were revealed in the time windows of the posterior P1 and N1 components prior to PCN (see Fig. 3). It might thus be argued that PCN in the current experiment was triggered in a pure bottom-up way based on the sensory asymmetry elicited by the lateralized retro-cue arrows. Future studies should try to focus on non-lateralized retro-cues (e.g. certain colors for indicating the cued information) for enabling a dissociation of posterior asymmetries related to sensory and attentional mechanisms. PCN was followed by a Pd effect that was strongest over lateral parieto-occipital areas (see Fig. 3). This component was shown to reflect active inhibitory mechanisms required for the re-allocation of attention after capture toward distracting stimuli^28^. Although the retro-cue in the present study was relevant for solving the task, the attentional orienting caused by the arrows pointing to the left or right side of fixation had to be inhibited to enable efficient processing of the non-lateralized probe stimuli. Pd can therefore be interpreted as an indicator for the closure of spatial attentional orienting related to the retro-cue that is independent from the updating of representations in visuo-spatial WM. Additionally, there was a more anterior contralateral negativity over lateral pre-motor areas that overlapped with PCN but was strongest at about 350–400 ms following the retro-cue (see Fig. 4). This effect corresponds to anterior directing attention negativity (ADAN) that can be observed in studies with attentional cues preceding the imperative information. ADAN was associated with supramodal control over the orienting of spatial attention^25^,^26^,^27^. The occurrence of this component in the current study can thus be interpreted as a top-down mechanism for shifting attention toward the cued mental representations.

Further information on the time course of visuo-spatial WM updating is provided by the probe-locked ERPs. Figure 6 shows an increased negativity in the non-cued compared to the new probe condition over frontal areas starting at about 400 ms. This effect can be linked to the N450 component that was revealed as a correlate of conflict processing and resolution in other cognitive tasks (e.g. in the Stroop task^24^). In the current experiment, it followed an increased positivity for the non-cued probe condition compared to the new probe condition that was associated with familiarity-driven recognition concerning the non-cued information^17^. Interestingly, the N450 effect revealed a decline with increasing SOA that resembled the respective effect on behavioral level (cf. Figs 2C and 6). This SOA-dependent course of the N450 effect was also supported by the randomization tests. Only for the 1800 ms SOA condition, there was no longer a significant N450 difference between the non-cued and new probe condition. These results indicate that the time available for WM updating affects the extent of conflict in the non-cued referred to the new probe condition. Both the RT difference and N450 difference showed the strongest decline between the 400 ms and 600 ms SOA suggesting that updating of visuo-spatial WM contents proceeds within this time window. This is further supported by the time course of the contralateral negativities following the retro-cue that were associated with the selection of the cued information (or inhibition of the non-cued contents). On average, both the PCN and ADAN effects lasted for about 450 ms after retro-cue onset (see Figs 3 and 4). Thus the updating of visuo-spatial WM was not fully completed by the time the probe was presented in the 300 ms and 400 ms conditions. Yet in conditions when there was more time available, making use of the retro-cue to drop the non-cued contents from WM reduced the processing conflict in the non-cued probe condition.

Prior studies already indicated a decay of non-cued mental representations over time following a retro-cue^13^. The present study extents these findings and shows that it takes about 500 ms to select cued information by means of a retro-cue and to drop the no longer required information from WM. This is in line with a recent study by von Moorselaar *et al*.^39^ that investigated the time course of the retro-cue ability to protect a mental representation from perceptual interference. Comparable to the current data on the fate of non-cued mental representations, the authors showed that the protective function of retro-active attentional focusing is fully established after about 500 ms. Together, these findings indicate that the retro-cue induced protection from decay and protection from interference in a WM task have similar temporal characteristics.

A further decrease of the RT difference between the non-cued and new probe conditions was observed between the 600/1000 ms and the 1800 ms SOA conditions. Also the N450 difference further declined in the 1000 ms and 1800 ms SOA conditions. This points toward a continuous decay of the representational strength of the non-cued information when the cued WM representations had already been selected for maintenance following the retro-cue^13^. Yet, there was still a difference in RTs when processing non-cued compared to new probes in the 1800 ms SOA condition. Thus while the cued or attended mental representations are actively maintained in WM, there is an additional spatial store that retains non-attended information long enough to modulate performance after the 1800 ms retention interval. This corresponds to current theories on fragile vSTM that can store non-attended or passive information longer than iconic memory (i.e. longer than 500 ms)^14^,^15^,^16^. By means of experimental paradigms with multiple retro-cues it was shown that attention can be re-oriented toward information in this short-term store^15^. This is in line with the current findings indicating that non-attended information in fragile vSTM can still affect performance in an ongoing task. Further experiments should concentrate on retention intervals longer than the 1800 ms used here to find out more about the temporal limits of fragile vSTM representations in a retro-cuing task.

So far, PCN and ADAN were associated with the selection of the cued mental representation for further retention in visuo-spatial WM. However, an electrophysiological parameter that constitutes a correlate of the active retention of relevant information following the retro-cue has to be sustained throughout the varying retention intervals. Such an effect was not shown on ERP level (see Figs 3 and 4). We therefore further concentrated on induced alpha lateralizations following the retro-cue and could reveal a sustained posterior decrease or desynchronization in alpha power contralateral to retro-cue direction (see Fig. 5). However, the result pattern in the 1800 ms condition was not entirely clear regarding this effect. While a sustained posterior alpha power asymmetry is evident in Fig. 5, this effect was rather small (d = −0.4) in the late phase of the retention interval (i.e. 1400–1800 ms). This might be explained by the fact that the current task does not necessarily require a sustained spatial representation of the cued color item following the retro-cue. While some participants retained the spatial code in WM, others might have focused on the non-spatial color information when the retention interval between retro-cue and probe display was long enough. Also prior studies suggested that the likelihood that participants strategically re-encode visuo-spatial information into non-spatial verbal codes increases beyond retention intervals of 1000 ms^40^,^41^. According to this assumption, the alpha asymmetry persisted in the retention intervals of the 600 ms and 1000 ms SOA conditions.

As the current experiment was based on non-lateralized probe displays, this posterior alpha effect cannot be related to a sustained orienting of attention toward the to be probed stimulus location. Fukuda *et al*.^42^ investigated the role of posterior alpha power asymmetry in a pre-cue design. The authors showed that the suppression of contralateral alpha power is linked to the selective retention of lateralized relevant information in WM and not to the inhibition of irrelevant signals (see^43^,^44^,^45^,^46^). Accordingly, the current posterior alpha asymmetry during the retention interval following a retro-cue can be associated with the selective retention of the cued mental representations in visuo-spatial WM. On the contrary, Myers *et al*.^22^ suggested that the contralateral alpha suppression reflects a transient access on the mental representations indicated by a retro-cue. However, this study always included a bilateral stimulus array following the retro-cue. It is therefore impossible to judge if the posterior asymmetry in alpha power reflected a transient mechanism or if it was simply cancelled by the subsequent perceptual inputs (like it was cancelled by the onset of the probe display in the current study; see Fig. 5).

In conclusion, the present study revealed the temporal progress of retro-cue induced WM updating. In a first phase, the cued mental representations have to be selected for further maintenance in WM. We suggest that the retro-cue locked PCN/ADAN complex is a correlate of this selection process. While PCN reflected the orienting of attention in retro-cue direction, ADAN was associated with a higher-level mechanism for controlling the allocation of attention toward the cued mental representations. We furthermore revealed a sustained decrease in induced alpha power contralateral to retro-cue direction. This effect was associated with the retention of the mental representation selected after the retro-cue. Yet these electrophysiological parameters only give information about the attentional focusing on the cued information but not about the fate of the non-cued mental representations. This was investigated by comparing performance and ERPs in the non-cued and new probe conditions for the different SOAs between retro-cue and probe display. The RT difference and N450 difference (non-cued minus new) featured a comparable decline with increasing SOA and both revealed the strongest change in the probe condition effect between the 400 and 600 ms SOA conditions. This suggests that WM updating was not fully completed when the probe display was already presented 300 ms or 400 ms following the retro-cue. Thus the non-cued information was still represented in WM and led to a greater conflict in probe processing. This conflict decreased even more in the 1800 ms SOA condition suggesting a continuous decay of the non-cued mental representation when attention was engaged otherwise^13^,^16^. Therefore, the current results support the notion that visuo-spatial WM corresponds to attended mental representations while encoded but no longer attended inputs are transferred to more passive and fragile vSTM representations within about 500 ms. Compared to attended inputs in WM, these representations in fragile vSTM revealed a lower impact on ongoing information processing.

## Methods

### Participants

Twenty participants took part in the experiment. One participant had to be excluded from data analysis, because error rates were particularly high in the cued probe condition (>50% errors). Thus data of nine female and ten male participants were used for final analyses (M(age) = 24.95 years; SD(age) = 2.12). All participants were right-handers. As shown by means of a screening questionnaire, no neurological or psychiatric diseases were reported. All participants had normal or corrected-to-normal vision. Participation was rewarded by course credit or a payment of 8 € per hour. Prior to beginning the experiment, all participants provided informed written consent. The study was approved by the ethic committee of the Leibniz Research Centre for Working Environment and Human Factors and was conducted in accordance with the Declaration of Helsinki.

### Stimuli and procedure

**Figure 1:**
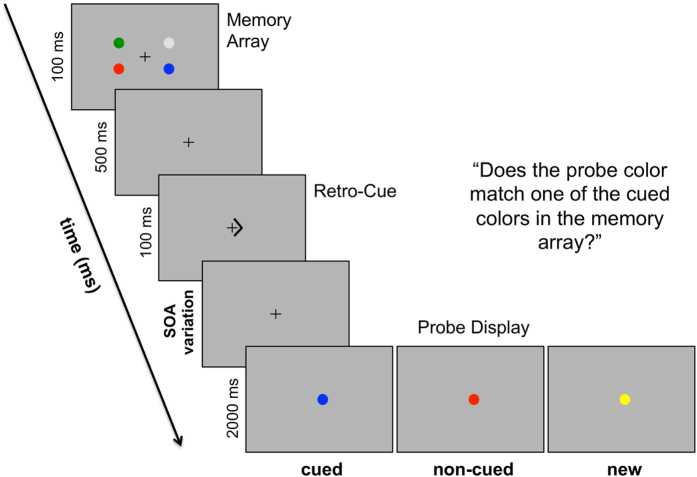
The experimental design used in the current study. Participants were instructed to remember which color was presented at the four different locations in the memory array. A retro-cue (left vs. right arrow) was presented 600 ms after memory array onset and indicated the memorized items that were required for the later recognition task. After the retro-cue, there was a retention interval varying in five steps (i.e. five SOA conditions: 300, 400, 600, 1000 and 1800 ms). The following probe display either contained a cued probe, a non-cued probe or a new probe (i.e. a stimulus not included in the initial memory array). Participants had to indicate by button-press whether the color of the probe stimulus matched the color of a cued memory array item.

**Figure 2:**
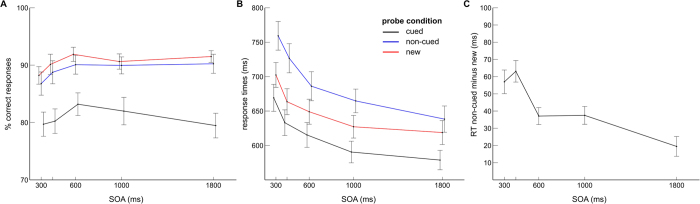
Accuracy (**A**) and response times (RTs; **B,C**) dependent on probe condition and SOA. Accuracy was lowest in the cued probe condition (black). Furthermore, a higher accuracy was shown for the new probe condition (red) compared to the non-cued probe condition (blue). (**B**) depicts RTs for the three probe conditions and five SOA conditions. The RT differences between the non-cued probe condition and new probe condition is shown in (**C**). Increased RT differences were revealed for the 300 ms and 400 ms SOA conditions compared to the remaining SOA conditions. Error bars depict the standard error of the mean.

The experiment was run on a 22-inch CRT monitor (100 Hz) that was set up with a viewing distance of 120 cm. Stimulus presentation was controlled by a VSG 2/5 graphic accelerator (Cambridge Research Systems, Rochester, UK). The background luminance was 10 cd/m^2^. The first stimulus display was a memory array with four differently colored circles presented left and right of central fixation for 100 ms with 25 cd/m^2^ each. The colors of these circles were randomly drawn from a list of eight colors (green, red, yellow, orange, pink, blue, petrol/blue, gray). Following the memory array, an arrowhead retro-cue appeared with an SOA of 600 ms and pointed randomly to the left or right side of the prior memory array. The retro-cue was also shown for 100 ms. It indicated whether the two memory items on the left or right side of fixation were relevant for the task in the current trial. Five different SOAs were used for the further delay between retro-cue and probe display (300, 400, 600, 1000, 1800 ms). The probe could either be displayed in a color previously shown on the cued side of the memory array (cued probe condition; 50% of trials) or on the non-cued side (non-cued probe condition; 25% of trials). Additionally, the probe color could be new with respect to the previous memory array (new probe condition; 25% of trials). The new color was randomly drawn from the four colors remaining after color assignment for the memory array. The probes were displayed for 2000 ms, irrespective of the time of response onset (see Fig. 1). Participants were instructed to indicate by button press whether the probe color was presented on the cued memory array side. Therefore, they were also told that the non-cued memory items were not required for solving the task. No further information was given about the different probe conditions that could appear in the course of the experiment. The task implied a YES response for the cued probe condition and a NO response for the non-cued and new probe conditions. Thus the proportion of trials requiring YES vs. NO responses was even. The assignment of YES and NO responses to response keys was varied between participants. Participants were instructed to respond as fast and accurate as possible.

Force keys were used for response measurement. Exerted force was directly recorded along with the raw EEG signal and an online analysis of force values enabled a performance overview during the experiment. We used a segmented regression procedure for offline detection of response onsets^47^. Trigger for left vs. right responses were set at force onset by means of EEGLAB^48^ for Matlab® (Mathworks Inc., Natick, MA). The experiment was divided into 12 blocks with 160 trials each. A two-minute break followed after each block to prevent confounding effects of fatigue during the experiment.

### Data analysis

#### Behavioral data

Errors in the current experiment involved missed responses (no response within 1400 ms after probe presentation) and incorrect assignments of YES vs. NO responses. All responses prior to 150 ms after probe display onset were categorized as “fast guesses” and were also treated as errors. The average rate of fast guesses was 0.088%. Fast guesses did not differ between the three probe conditions or between YES and NO responses. To test for differences in response accuracy and RTs separated ANOVAs with the within-subject factors “probe condition” (cued, non-cued, new) and “SOA” (300, 400, 600, 1000, 1800 ms) were conducted. We applied Greenhouse-Geisser correction when sphericity of the data was violated. Greenhouse-Geisser ε is given in these cases. Benjamini-Hochberg correction was used for post-hoc pairwise comparisons^49^. Additionally, we investigated if the hypothesized RT difference between the non-cued and new probe conditions declined with an increasing retention interval following the retro-cue. Therefore, an ANOVA was calculated with the RT difference as dependent variable and SOA as a continual/numeric factor. Partial eta squared (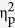) is reported for all ANOVAs concerning behavioral and EEG data.

#### EEG recording and processing

EEG was recorded from 60 Ag/AgCl active electrodes (ActiCap; Brain Products, Gilching, Germany) that were affixed across the scalp according to the extended 10/20 System^50^. Eye movements were measured by means of two electrode pairs applied above/below the left eye (vertical EOG) and at the outer canthi of each eye (horizontal EOG). A BrainAmp DC-amplifier sampled EEG and EOG with a frequency of 1000 Hz. A 250 Hz low-pass filter was used during recording. Midline electrode FPz served as the ground electrode. Electrode impedance was kept below 5 kΩ during recording.

EEGLAB, ERPLAB^51^ and MATLAB® were used for further EEG analyses. Data were re-referenced offline to the averaged mastoids by means of the signal recorded from electrodes TP9 and TP10. The data were divided into segments ranging from 500 ms before to 4000 ms after presentation of the memory array. The 200 ms interval preceding the memory array served as baseline. EEG data were 0.5 Hz high-pass (6601 point FIR filter; transition band width 0.5 Hz; cut-off frequency 0.25 Hz) and 30 Hz low-pass filtered (441 point FIR filter; transition band width 7.5 Hz; cut-off frequency 33.75 Hz). Independent component analysis (ICA) was used to semi-automatically correct for eye blinks and generic data discontinuities. Every fourth trial served as the basis for ICA. Artifacted ICs were detected by means of ADJUST^52^. Segments with remaining artifacts were removed through automatic epoch rejection implemented in EEGLAB. Only correct trials were used for further analyses.

#### ERP data

ERP data were studied referred to the onset of the retro-cue and probe display. In the first place, the contralateral and ipsilateral portions of the ERP locked to the retro-cue were analyzed according to retro-cue direction (left vs. right). Comparable to techniques already applied for parameterizing selective attentional orienting to perceptual information^28^, we quantified attentional processing following the retro-cue by means of the negative area under the contralateral vs. ipsilateral difference wave in a time window from 200–500 ms at electrodes PO7/8 (i.e. posterior contralateral negativity; PCN). Pd was analyzed by means of the positive area under the difference wave from 400–700 ms after retro-cue presentation at electrodes PO7/8. ADAN was investigated by means of the negative area under the contralateral minus ipsilateral difference wave from 200–500 ms after retro-cue onset at electrodes FC3/4. Only the 600 ms, 1000 ms and 1800 ms SOA conditions were used for the PCN, ADAN and Pd analyses, because there was a strong overlap of retro-cue and probe processing in the shorter SOA conditions.

**Figure 3:**
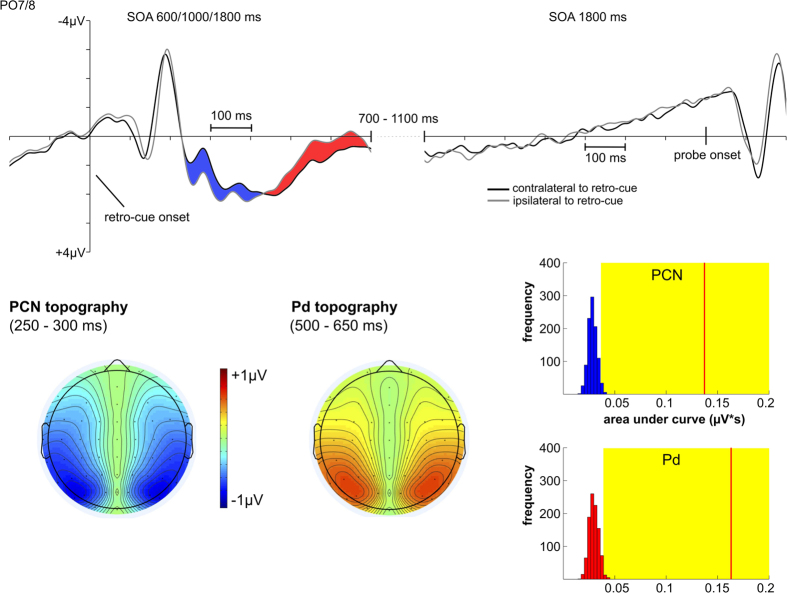
Posterior ERPs (PO7/8) dependent on retro-cue direction. In the upper row, the contralateral and ipsilateral portions of the posterior ERP are depicted for the 600, 1000 and 1800 ms SOA conditions for a 700 ms interval following the retro-cue. The area marked in blue corresponds to PCN. The red area corresponds to the Pd effect. Additionally, a later time window from 1300–2000 ms after the retro-cue is shown for the 1800 ms SOA condition. PCN and Pd topographies revealed maxima over lateral parieto-occipital areas. On the bottom right, the results of randomization tests in the PCN interval (200–500 ms) and Pd interval (400–700 ms) are shown. The blue (PCN) and red (Pd) bars indicate the random distribution resulting from 1000 permutations with the frequency of measured values denoted on the y-axis and the respective area values denoted on the x-axis. The red lines represent the grand average values of the negative and positive areas observed for PCN and Pd. The yellow areas indicate the top 5% of the permutation distributions. Red lines falling within the yellow areas indicate the significant PCN and Pd effects.

**Figure 4:**
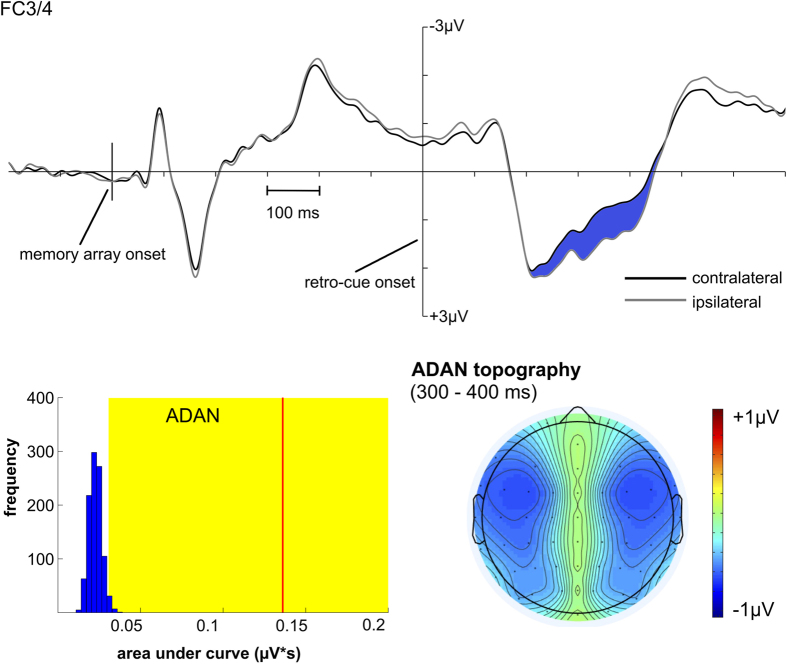
Anterior ERPs (FC3/4) dependent on retro-cue direction. The contralateral and ipsilateral portions of the ERP were averaged for the 600, 1000 and 1800 ms conditions. ADAN corresponds to the blue area marked (contralateral minus ipsilateral) from 200–500 ms following the retro-cue. The randomization tests for the negative area in this time interval and the anterior ADAN topography are shown in the lower row. Concerning the randomization test, the bars indicate the random distribution resulting from 1000 permutations with the frequency of measured values denoted on the y-axis and the respective area values denoted on the x-axis. The red line represents the grandaverage value of the negative ADAN area. The yellow areas indicate the top 5% of the permutation distribution. The ADAN effect is statistically significant, as the red line falls within the yellow area. The anterior topography of the ADAN effect is shown on the bottom right.

As the area measures only include non-zero values, noise in the data will always bias the area toward values greater than zero. This requires a special procedure to test if the measured areas actually differ from chance level^28^. In order to measure the negative or positive area under a contralateral - ipsilateral difference wave only produced by noise, we randomly assigned left and right retro-cue conditions to the trials. Then the negative area (200–500 ms) and positive area (400–700 ms) were measured and averaged across participants. This procedure was run 1000 times resulting in a random distribution based on the varying assignments of left and right retro-cue conditions. If the area value measured in the original data was higher than 95% of this random distribution, it was considered to differ significantly from chance (see Figs 3 and 4).

The analyses of the probe-locked ERPs focused on non-lateralized effects between the non-cued and new probe condition (see Fig. 6). We calculated the difference of the ERP in the non-cued and new conditions. According to prior results from Schneider *et al*.^17^ based on a comparable experiment design, the N450 effect was measured by means of the negative area under this difference curve in an interval from 400–600 ms after probe onset. An electrode cluster comprising left-frontal (FC1, F3) and midline-frontal electrodes (FCz, Fz) was used for this analysis. Comparable to the behavioral analyses, an ANOVA was calculated treating the different SOAs as numeric values to test for a temporal decline of the N450 difference with increasing SOA. Additionally, we also ran the randomization analyses based on the non-cued minus new difference curves. Therefore, the non-cued and new probe conditions were randomly assigned to each trial within an SOA condition. Then the negative area (400–600 ms) under the non-cued minus new difference wave was measured and averaged across participants. After running this procedure 1000 times, we compared the measured N450 difference for each SOA with the 95% and 90% threshold values resulting from the 1000 permutations (see Fig. 6).

#### Time-frequency data

**Figure 5:**
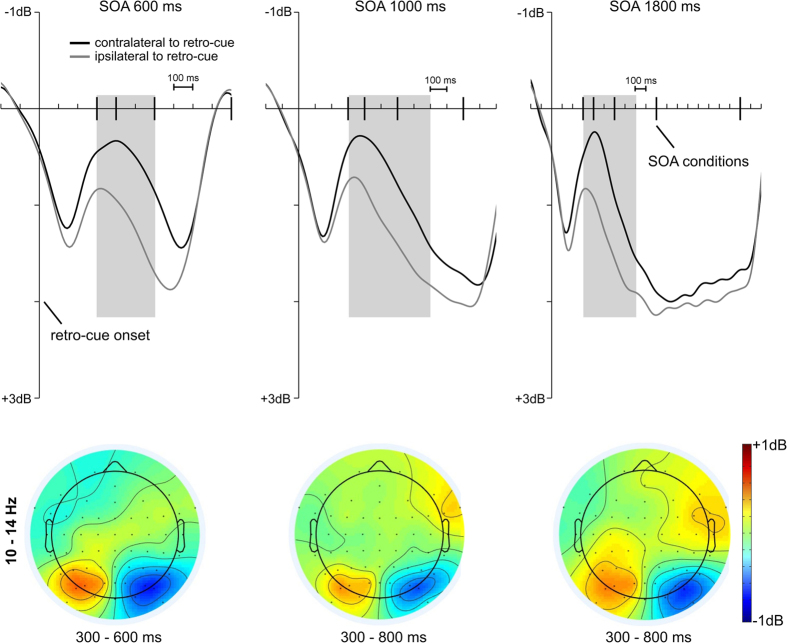
Posterior asymmetries (PO7/8) in induced alpha power (10–14 Hz). The upper row shows the contralateral and ipsilateral portions of induced alpha power referred to retro-cue direction for the 600, 1000 and 1800 ms SOA conditions. The grey area marks the interval used for the topographic maps. The topographies for left minus right retro-cue conditions are shown in the lower row and indicate a decrease in contralateral vs. ipsilateral alpha power over lateral parieto-occipital areas for all SOA conditions.

**Figure 6:**
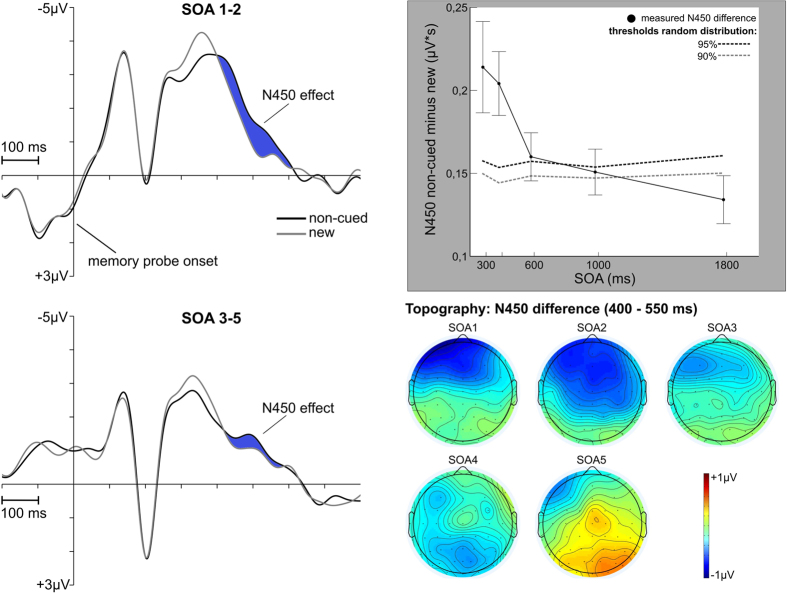
Frontal ERPs time-locked to probe display onset. On the left side, the ERPs at a left-frontal and midline-frontal electrode cluster (FCz, FC1, Fz, F3) are shown averaged for the 300 and 400 ms SOA conditions (upper graph) and the 600, 1000 and 1800 ms SOA conditions (lower graph). Furthermore, the graph highlighted in grey shows a decline of frontal negative difference with increasing SOA. While the x-axis of this graph denotes the 5 SOA conditions, the y-axis denotes the respective area values (in μV * s). Error bars depict the standard error of the mean. The gray and black dotted lines depict the 95% and 90% threshold values resulting from the randomization tests for each SOA condition. The decline of the N450 difference is also evident on topographic level.

Spectral power was computed by convolving 3-cycle complex Morlet wavelets with each epoch of the EEG data. The number of cycles in the wavelets used for higher frequencies expanded with a factor of 0.5. Epochs consisted of 200 time points between −282 ms and 2082 ms referred to retro-cue onset. The frequencies used for this computation ranged from 4 Hz to 20 Hz in 32 logarithmic steps. The 200 ms interval preceding the memory array served as baseline. Lateralized effects in induced oscillations related to retro-cue side were revealed in the upper alpha range (10–14 Hz)^17^. Contralateral and ipsilateral induced alpha power was thus measured in the 10–14 Hz range for the 600 ms, 1000 ms and 1800 ms SOA conditions (see Fig. 5). We used T-tests against zero based on the contralateral minus ipsilateral power difference in the 10–14 Hz range to test for a decrease in contralateral compared to ipsilateral alpha power. Separate analyses were run for the three SOA conditions. While mean alpha power was measured between 300–1000 ms in the 1000 ms and 1800 ms SOA conditions, this time window was reduced to 300–600 ms in the 600 ms SOA condition. This was done to exclude that lateralized effects found in the 600 ms SOA condition could be modulated by probe processing. Additionally, a late mean alpha power time window from 1400–1800 ms was used in the 1800 ms SOA condition. T values, p values (corrected for multiple testing by means of false discovery rate or FDR correction^49^) and Cohen’s d are reported for all t-tests.

## Additional Information

**How to cite this article**: Schneider, D. *et al*. The time course of visuo-spatial working memory updating revealed by a retro-cuing paradigm. *Sci. Rep.* **6**, 21442; doi: 10.1038/srep21442 (2016).

## Supplementary Material

Supplementary Information
