# Supplementary material for: The time course of visuo-spatial working memory updating revealed by a retro-cuing paradigm
Source: Sci Rep. 2016 Feb 12;6:21442. doi: 10.1038/srep21442 (PMC4751472; doi:10.1038/srep21442)
Supplement: Supplementary Information [file srep21442-s1.pdf]

Supplementary figures

The time course of visuo-spatial working memory updating revealed by a retro-cuing paradigm

Daniel Schneider*, Christine Mertes & Edmund Wascher

Leibniz Research Centre of Working Environment and Human Factors, TU Dortmund

Supplementary figure S1: The figure shows posterior event-related lateralizations (ERLs) time-locked to the central probe stimulus. ERLs were calculated by subtracting the contralateral from the ipsilateral portion of the ERP after probe onset referred to retro-cue direction (left vs. right). Sustained posterior contralateral negativity (SPCN) appeared in all probe conditions and followed early sensory asymmetries in the P1 and N1 range. These early sensory effects can be explained by the fact that the retro-cue led to a shift of spatial attention in cue direction. Especially in the short SOA conditions, attention was still shifted in cue direction when the probe was presented, leading to posterior P1 and N1 asymmetries. Most notably, SPCN was evident in all probe conditions. This indicates that performance in all probe condition was based on the access on the cued memory array contents. The non-cued probe condition was not solved by a re-focusing on the non-cued memory array information. As SPCN duration usually co-varies with response times, the duration of the SPCN effect also differed between probe conditions in the current experiment.

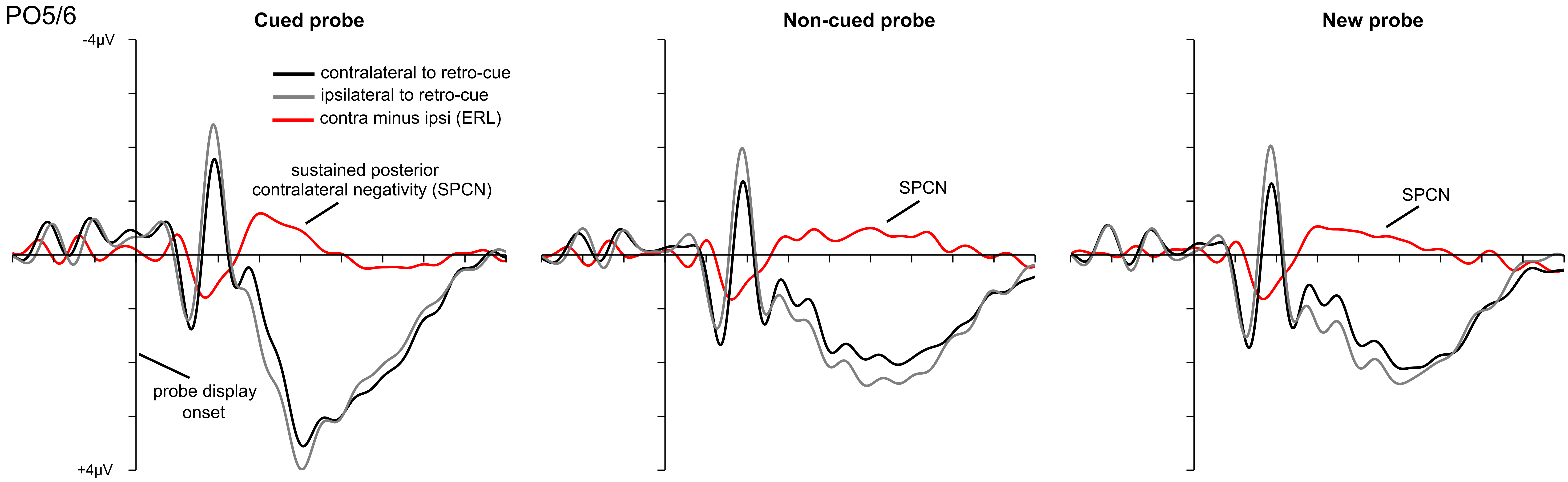
PO5/6
Cued probe
Non-cued probe
New probe
-4µV
+4µV
contralateral to retro-cue
ipsilateral to retro-cue
contra minus ipsi (ERL)
sustained posterior
contralateral negativity (SPCN)
probe display
onset
SPCN
SPCN
